# Peroxisome Proliferator-Activated Receptor *α* Reduces Endothelin-1-Caused Cardiomyocyte Hypertrophy by Inhibiting Nuclear Factor-*κ*B and Adiponectin

**DOI:** 10.1155/2016/5609121

**Published:** 2016-10-11

**Authors:** Hsu-Lung Jen, Po-Len Liu, Yung-Hsiang Chen, Wei-Hsian Yin, Jaw-Wen Chen, Shing-Jong Lin

**Affiliations:** ^1^Division of Cardiology, Cheng-Hsin General Hospital, Taipei, Taiwan; ^2^Institute of Clinical Medicine, Faculty of Medicine, Institute of Pharmacology, and Cardiovascular Research Centre, National Yang-Ming University, Taipei, Taiwan; ^3^Department of Respiratory Therapy, College of Medicine, Kaohsiung Medical University, Kaohsiung, Taiwan; ^4^Graduate Institute of Integrated Medicine, College of Chinese Medicine, China Medical University, Taichung, Taiwan; ^5^Department of Psychology, College of Medical and Health Science, Asia University, Taichung, Taiwan; ^6^Department of Medical Research, Division of Cardiology, Department of Medicine, Taipei Veterans General Hospital, Taipei, Taiwan

## Abstract

Peroxisome proliferator-activated receptor *α* (PPAR*α*) plays a role in the pathogenesis of cardiac hypertrophy, although its underlying mechanism remains unclear. The purpose of this study was to evaluate the effect of PPAR*α* activation on endothelin-1- (ET-1-) caused cardiomyocyte hypertrophy and explore its underlying mechanisms. Human cardiomyocytes (HCMs) were cultured with or without ET-1, whereafter the inhibitory effects of fenofibrate, a PPAR*α* activator, on cell size and adiponectin protein were tested. We examined the activation of extracellular signal-regulated kinase (ERK) and p38 proteins caused by ET-1 and the inhibition of the ERK and p38 pathways on ET-1-induced cell size and adiponectin expression. Moreover, we investigated the interaction of PPAR*α* with adiponectin and nuclear factor-*κ*B (NF-*κ*B) by electrophoretic mobility shift assays and coimmunoprecipitation. ET-1 treatment significantly increased cell size, suppressed PPAR*α* expression, and enhanced the expression of adiponectin. Pretreatment with fenofibrate inhibited the increase in cell size and enhancement of adiponectin expression. ET-1 significantly activated the ERK and p38 pathways, whereas PD98059 and SB205380, respectively, inhibited them. Our results suggest that activated PPAR*α* can decrease activation of adiponectin and NF-*κ*B and inhibit ET-1-induced cardiomyocyte hypertrophy.

## 1. Introduction

Cardiac hypertrophy is an adaptive response of the heart that preserves cardiac pump function under adverse conditions, and prolonged hypertrophy is one of the major predictors of heart failure [1, 2]. Previous studies demonstrated that endothelin-1 (ET-1) plays a critical role in the induction of myocyte hypertrophy [3, 4].

Peroxisome proliferator-activated receptor *α* (PPAR*α*) deficiency can impair the heart's functional capacity [5]. Activation of PPAR*α* reduces cardiac hypertrophy, decreases cardiac fibrosis, attenuates cardiac dysfunction, and improves survival by inhibition of profibrotic, proinflammatory, and prohypertrophic genes [6]. Fenofibrate, a PPAR*α* activator, has been reported to reduce ET-1-caused cardiac hypertrophy through downregulation of activating protein-1 (AP-1) binding and inhibition of p38 mitogen-activated protein kinases (MAPK) signaling [7, 8]. Fenofibrate-activated PPAR*α* can also interfere with ET-1-induced cardiomyocyte hypertrophy by modification of nuclear factor of activated T-cells- (NFAT-) related signals [9, 10]. Moreover, a statin was reported to inhibit cardiac hypertrophy by inhibiting negative cross talk between nuclear factor-*κ*B (NF-*κ*B) and PPAR*α* [11]. However, other possible molecular mechanisms related to cardiomyocyte hypertrophy by PPAR*α* modulation remain to be elucidated.

Adiponectin is an abundant serum adipokine derived from adipose tissue that exhibits protective effects in the myocardium and vasculature [12]. Adiponectin inhibits hypertrophic signals in the myocardium and contributes to the regulation of cardiac remodeling [13]. Moreover, adiponectin may significantly enhance PPAR*α* activity, and the adiponectin-dependent PPAR*α* activation may play a protective role against angiotensin II-induced cardiac fibrosis [14]. However, not all of the effects of adiponectin are beneficial if its circulating or tissue levels are incessantly elevated. For instance, adiponectin may activate NF-*κ*B and AP-1, resulting in expression of proinflammatory genes and enhancement of angiotensin II-induced cardiac fibroblast proliferation [15, 16], which may, in turn, play a role in heart failure progression [17, 18]. Administration of adiponectin can nevertheless increase energy expenditure and decrease body weight, which may be implicated in cardiac cachexia development in advanced heart failure [19, 20].

In our previous studies, we found that both ET-1 and angiotensin II acutely stimulated adiponectin expression and significantly increased cell size in cultured human cardiomyocytes (HCMs) [21]. Therefore, we hypothesized that such a link may also exist between PPAR*α*, NF-*κ*B, adiponectin, and cardiac hypertrophy. The aim of the present study was to clarify this hypothesis.

## 2. Materials and Methods

### 2.1. HCM Culture

HCMs were purchased from ScienCell Research Laboratories (Carlsbad, CA, USA) and grown in cardiac myocyte medium supplemented with 5% fetal bovine serum (FBS), 1% cardiac myocyte growth supplement, and 1% penicillin/streptomycin solution. Cells were cultured on a poly-L-lysine-coated dish in a humidified incubator with 5% CO_2_ at 37°C. Cells between passages 3–5 were used in all experiments. The Safety Committee of Biological Experiments at Kaohsiung Medical University approved the use of HCMs for* in vitro* experimental use. The following drugs were used: fenofibrate (a PPAR*α* agonist, Sigma), GW6471 (GW, PPAR*α* antagonist, Santa Cruz), BQ-123 [BQ123, endothelin A receptor (ETA) antagonist, Sigma], BQ788 [BQ788, endothelin B receptor (ETB) antagonist, Sigma], PD98059 (ERK inhibitor, Cell Signaling), and SB203580 (p38 inhibitor, Cell Signaling). None of these drugs significantly influence cell viability (>90%).

### 2.2. Measurement of Cell Surface Area

Rhodamine-phalloidin was used to visualize actin fibers under fluorescence microscopy. HCMs were stained with rhodamine-phalloidin (1 : 100 dilute, Millipore) to target F-actin in the cytosol and then stained with 4′,6-diamidino-2-phenylindole (DAPI) (1 : 10,000 dilution, Millipore) for 5 min to target DNA in the cell nucleus. HCMs were viewed under a camera attached to a microscope. The surface area was determined using image analysis software (MetaMorph Imaging System, Meta Imaging Series 4.5) and calculated as the mean of 100 cells from at least ten randomly chosen fields (×40 objective) in three separate experiments.

### 2.3. Western Blot

The cell lysates were prepared by addition of cell lysis buffer (Cell Signaling, Danvers, MA). Cell lysates were subjected to 12% sodium dodecyl sulfate-polyacrylamide gel electrophoresis (SDS-PAGE) and transferred onto polyvinylidene difluoride membranes for immunoblotting. Blots were incubated with various primary antibodies: rabbit-anti-human-adiponectin (Abcam, Cambridge, MA), rabbit-anti-human-beta-myosin heavy chain (*β*-MHC; Santa Cruz Biotechnology, CA), rabbit-anti-human-B-type natriuretic peptide (BNP; Santa Cruz Biotechnology, CA), goat-anti-human-ERK (Cell Signaling, Danvers, MA), rabbit-anti-human phosphor-p38 MAPK (Cell Signaling, Danvers, MA), rabbit-anti-human-p65 (Santa Cruz Biotechnology, CA), histone H1 (Santa Cruz Biotechnology, CA), and mouse-anti-human-*β*-actin (Sigma, St. Louis, MO) for 1 h. Signal was detected using Chemiluminescence Reagent Plus (NEN, Boston, MA) [22, 23]. The intensity of each band was quantified by a densitometer.

### 2.4. [^3^H] Leucine Incorporation

HCMs were cultured in 48-well dishes, pretreated with different drugs, and then stimulated with or without ET-1 (50 nM) and coincubated with [^3^H] leucine (1 *μ*Ci/mL) (Sigma, MA) for 48 h. Cells were washed with PBS and 10% trichloroacetic acid was added to the wells. Then, 10% trichloroacetic acid was removed and washed with 95% ethanol. After the wells had been dried, 1 mL of 0.5 mol/L NaOH was added to the wells and samples were transferred to scintillation vials to measure ^3^H-Leu incorporation.

### 2.5. Immunofluorescence Staining

HCMs were immunofluorescence stained with antibody (1 : 100 dilute, Abcam, Cambridge, MA) to target adiponectin and then stained with DAPI (1 : 10,000 dilution, Millipore) for 5 min to target DNA in the cell nucleus. HCMs were viewed under a camera attached to a microscope.

### 2.6. Electrophoretic Mobility Shift Assay (EMSA)

The NF-*κ*B probe was used for the gel shift assay, which was a 31-mer synthetic double-stranded oligonucleotide (5′-ACA AGG GAC TTT CCG CTG GGG ACT TTC CAG G-3′; 3′-TGT TCC CTG AAA GGC GAC CCC TGA AAG GTC C-5′) containing a direct repeat of the *κ*B site. For the EMSA, the digoxigenin (Dig) gel shift kit for 3′-end labeling of oligonucleotides (Roche, Indianapolis, IN, USA) was used for binding assays.

### 2.7. Coimmunoprecipitation Assay (Co-IP)

The nuclear protein from human cardiomyocytes was extracted by use of a nuclear protein extraction kit (Millipore, MA, USA). Monoclonal antibodies were added to 300 *μ*g of isolated nuclear protein and incubated in 4°C overnight. Then, 50 *μ*L of protein A/G-Sepharose bead suspension (Santa Cruz, CA) was added to each sample and gently mixed overnight. The samples were then centrifuged at 12,000 ×g for 30 s; thereafter, the beads were washed three times with 1 mL of IP buffer (Millipore, MA). The recovered beads were resuspended in SDS-PAGE loading buffer, and the supernatants were used for electrophoretic separation and immunoblotting.

### 2.8. Statistical Analyses

Data are presented as means ± SEM. Statistical differences between two groups were analyzed by unpaired Student's *t*-test, and differences between multiple groups of data were analyzed by one-way analysis of variance followed by a Bonferroni* post hoc* test with GraphPad Prism Software (Version 5.03). The criterion for statistical significance was *P* < 0.05.

## 3. Results

### 3.1. Fenofibrate Inhibited ET-1-Induced Cell Hypertrophy and Adiponectin Expression

**Figure 1:**
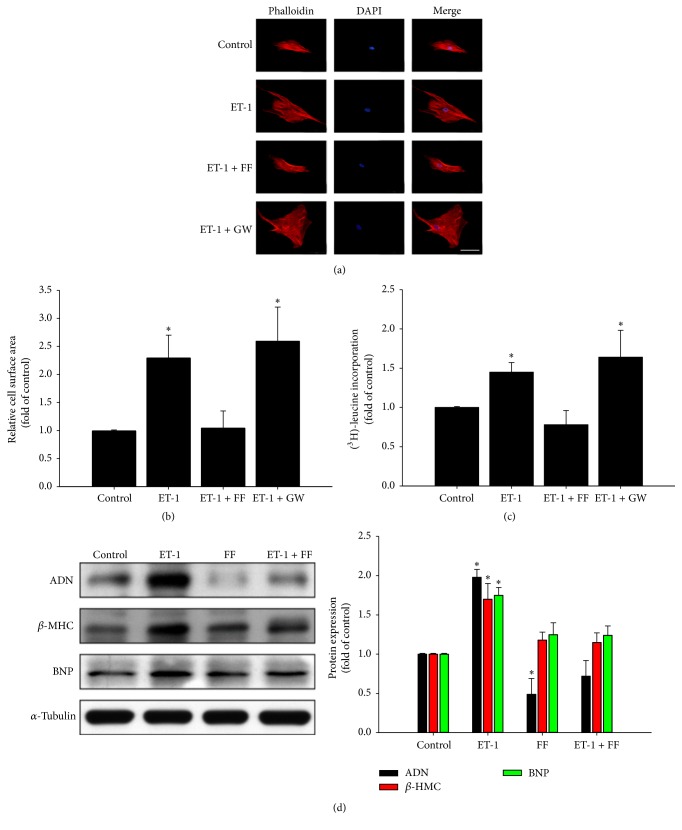
Fenofibrate inhibited ET-1-dependent hypertrophy and adiponectin expression. The role of fenofibrate and ET-1 in human cardiomyocyte cell size was assayed by pretreatment of cells with PPAR*α* agonist (fenofibrate; FF) or PPAR*α* antagonist (GW6471; GW) for 24 h, followed by stimulation with or without ET-1 (50 nM) for 48 h. Cells were fixed and stained with rhodamine-phalloidin (red) and DAPI (blue) followed by cell surface area quantization (scale bar, 50 *μ*m) (a). Data are expressed as mean ± SEM of values from three independent experiments (b). ^3^H-leucine incorporation was determined as a measure of relative protein synthesis (c). Western blot assay and quantification of adiponectin (ADN), *β*-myosin heavy chain (*β*-MHC), and B-type natriuretic peptide (BNP) expression were also demonstrated (d). ^*∗*^ *P* < 0.05, compared with untreated control group.

The roles of fenofibrate and ET-1 in HCM cell size were assessed by pretreatment of cells with a PPAR*α* agonist (fenofibrate) or PPAR*α* antagonist (GW6471) for 24 h and then stimulated with or without ET-1. As shown in Figures 1(a)–1(c), ET-1 significantly increased cell size and protein synthesis of cardiomyocytes, compared to that of the control group; but fenofibrate inhibited ET-1-caused cellular hypertrophy. Additionally, the use of ET-1 and PPAR*α* antagonist further increased cell size and protein synthesis of HCM. Moreover, immunoblot assay demonstrated that adiponectin expression was significantly enhanced in ET-1-treated HCMs compared to that in control HCMs. Furthermore, the enhancement of adiponectin expression by ET-1 in HCMs could be inhibited by pretreatment with fenofibrate (Figure 1(d)).

### 3.2. ETA Receptor Involved in ET-1-Induced Cell Hypertrophy and Adiponectin Expression

**Figure 2:**
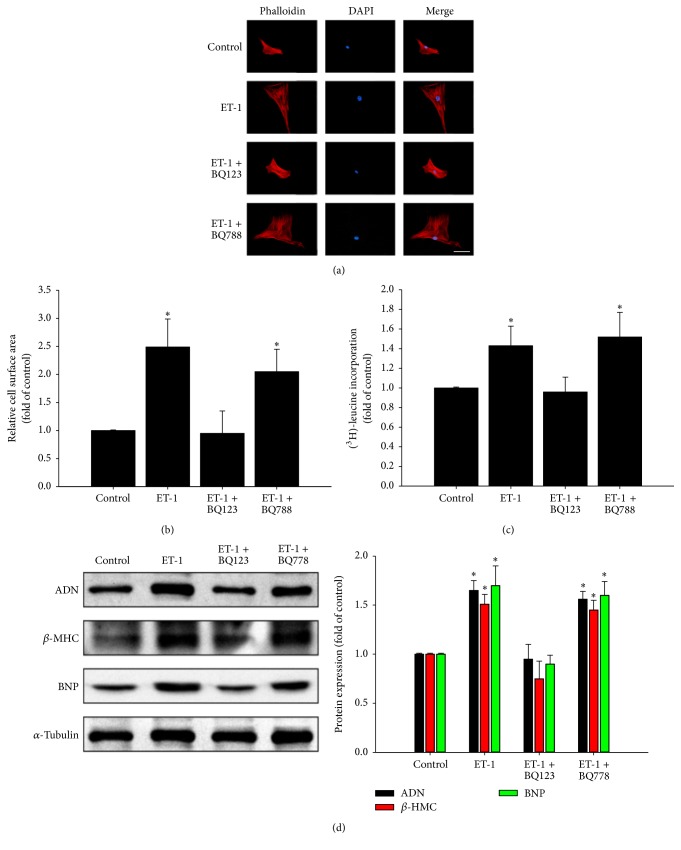
ETA receptor involved in ET-1-dependent hypertrophy and adiponectin expression. The role of ET-1 receptor and ET-1 in myocardial cell size was determined in cells pretreated with ETA receptor antagonist (BQ123) or ETB receptor antagonist (BQ788) for 1 h, followed by stimulation with or without ET-1 (50 nM) for 48 h. Cells were fixed and stained with rhodamine-phalloidin (red) and DAPI (blue) followed by cell surface area quantitation (scale bar, 50 *μ*m) (a). Data are expressed as mean ± SEM of values from three independent experiments (b). ^3^H-leucine incorporation was determined as a measure of relative protein synthesis (c). Immunoblot assay and quantification of adiponectin (AND), beta-myosin heavy chain (*β*-MHC), and B-type natriuretic peptide (BNP) expression were also demonstrated (d). ^*∗*^ *P* < 0.05, compared with untreated control group.

The role of ET-1 receptors in ET-1-induced cell hypertrophy and protein synthesis was studied in HCM cells pretreated with ETA receptor antagonist (BQ123) or ETB receptor antagonist (BQ788) for 1 h, followed by stimulation with or without ET-1 (50 nM) for 48 h. As shown in Figures 2(a)–2(c), pretreatment with the ETA receptor antagonist (BQ123), instead of the ETB receptor antagonist (BQ788), significantly abolished cell hypertrophy and protein synthesis of cardiomyocytes induced by ET-1. Moreover, the increased adiponectin expression induced by ET-1 was also mediated by the ETA receptor (Figure 2(d)).

### 3.3. Signal Transduction Pathways in ET-1-Induced Cell Hypertrophy and Adiponectin Expression

**Figure 3:**
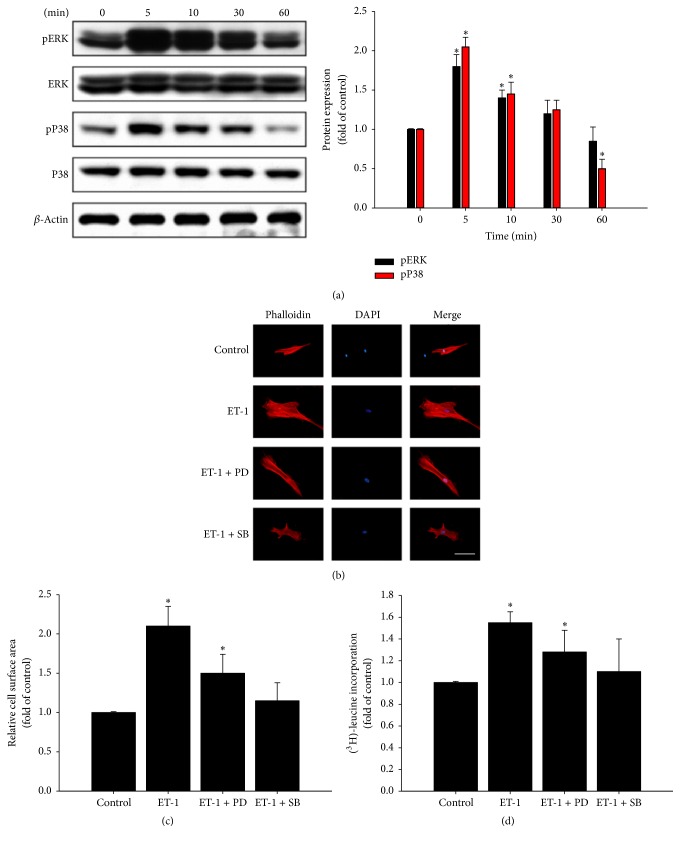
Signaling pathways in ET-1-induced cell hypertrophy and adiponectin expression. In human cardiomyocyte cells, phosphorylation of ERK and p38 was determined by immunoblot assay (a). Assays were performed in triplicate. The role of ERK and p38 and ET-1 in myocardial cell size was determined in cells pretreated with PD98059 (ERK inhibitor) or SB203580 (p38 inhibitor) for 1 h, followed by stimulation with or without ET-1 (50 nM) for 48 h. Cells were fixed and stained with rhodamine-phalloidin (red) and DAPI (blue) followed by cell surface area quantitation (scale bar, 50 *μ*m) (b). Data are expressed as mean ± SEM of values from three independent experiments (c). ^3^H-leucine incorporation was determined as a measure of relative protein synthesis (d). ^*∗*^ *P* < 0.05, compared with 0 min group (a) or untreated control group (c).

ET-1 significantly increased the cell size and adiponectin protein expression in HCM compared with that in the control group. As shown in Figure 3(a), phosphorylated ERK and p38 proteins were induced by ET-1 in a time-dependent manner. The increases in ET-1-induced cell hypertrophy and adiponectin protein expression were significantly, though only partially, attenuated after the addition of PD98059 or SB203580, but before ET-1 stimulation (Figures 3(b)–3(d)). The results suggest that ET-1 increases cell size and adiponectin protein expression through both ERK and p38 signaling pathways.

### 3.4. Fenofibrate Attenuated ET-1-Activated Adiponectin and NF-*κ*B

**Figure 4:**
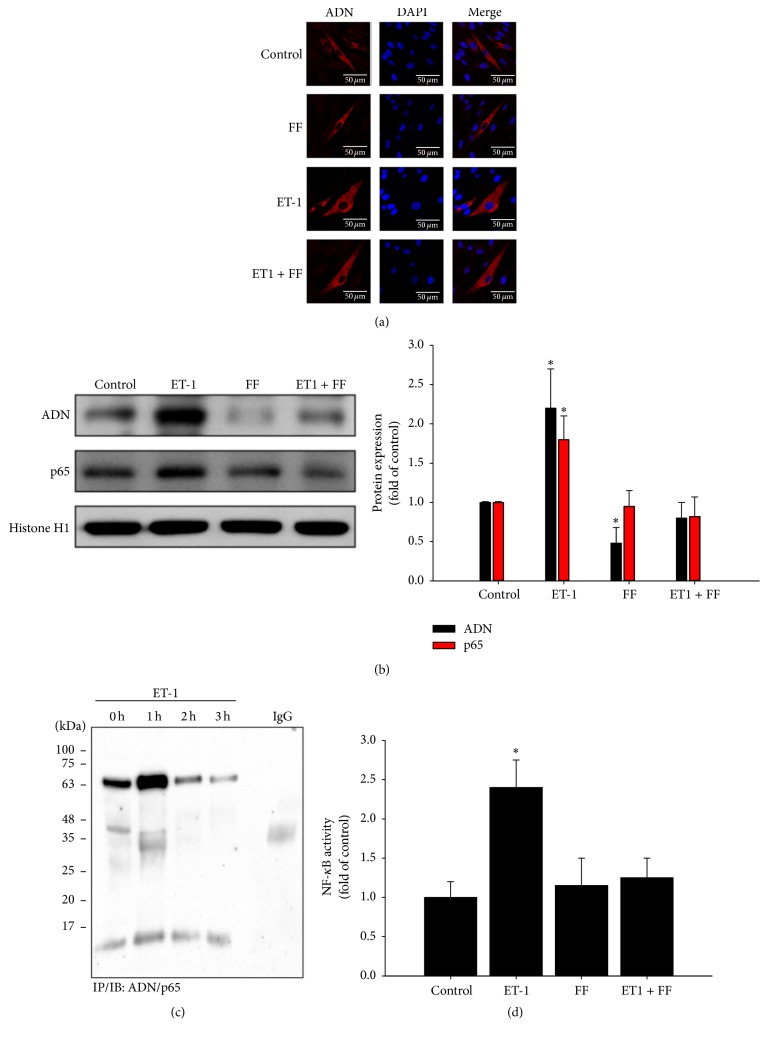
Fenofibrate attenuated ET-1-induced adiponectin and NF-*κ*B activation and DNA binding activity in cultured human cardiomyocytes. Immunofluorescence staining showed that pretreatment of HCMs with fenofibrate significantly inhibited ET-1-induced activation of adiponectin (a). The pretreatment of cells with fenofibrate (FF) inhibits adiponectin (ADN) and p65-NF*κ*B. Histone H1 was used as a loading control for nuclear protein (b). Fenofibrate (FF) attenuates ET-1-stimulated NF-*κ*B binding activity by EMSA assay (c). The summarized data (mean ± SEM) from 3 separate experiments were shown in the bar graph (d). ^*∗*^ *P* < 0.05, compared to untreated control group.

Immunofluorescence staining showed that pretreatment of HCMs with fenofibrate significantly inhibited ET-1-induced activation of adiponectin (Figure 4(a)). Pretreatment of HCMs with fenofibrate also significantly inhibited p65 protein nuclear translocation. Histone H1 was used as a loading control for nuclear proteins (Figure 4(b)). The EMSA also demonstrated that fenofibrate significantly attenuated ET-1-induced NF-*κ*B binding activity (Figure 4(c)). The summarized data from three independent experiments is shown in Figure 4(d).

### 3.5. Effects of ET-1 on the Association of Adiponectin, NF-*κ*B, and PPAR*α*

**Figure 5:**
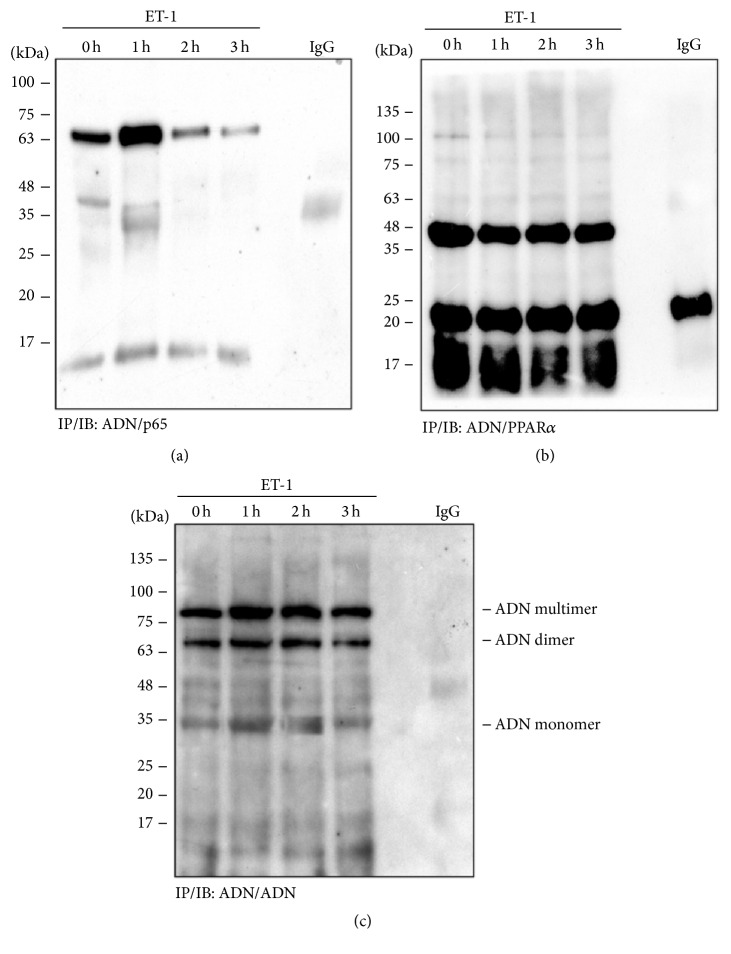
Effects of ET-1 on the association of adiponectin, NF-*κ*B, and PPAR*α* in nuclei of cultured human cardiomyocytes. Nuclear proteins extracted from cells were immunoprecipitated with antibodies against adiponectin (ADN) or control mouse IgG and then separated by 12% SDS-PAGE gels before immunoblotting with antibodies against p65 (a), PPAR*α* (b), and adiponectin (ADN) (c). The results were obtained from three independent experiments.

Proteins were isolated from cells and immunoprecipitated with antibodies against adiponectin or control mouse IgG and then probed with antibodies against p65 (Figure 5(a)), PPAR*α* (Figure 5(b)), and adiponectin (Figure 5(c)) and analyzed by immunoblotting. The results demonstrated that incubation of cells for 1 h with ET-1 significantly enhanced adiponectin and IP of adiponectin with p65, but not of adiponectin with PPAR*α*. Therefore, the PPAR*α* activation by fenofibrate may counteract the effects of ET-1 by preventing both adiponectin and NF-*κ*B.

## 4. Discussion

In this study, the functional relationships between PPAR*α* activation and ET-1-induced adiponectin expression and cell hypertrophy in cultured HCM were investigated* in vitro*. Several new observations were made in our study. ET-1 acutely increased cell size and stimulated adiponectin expression through the ETA receptor in HCM, probably mediated by activating the ERK and p38 pathways. Additionally, such an increase in cell size and enhancement of adiponectin expression were reduced by pretreatment with the PPAR*α* activator fenofibrate. Lastly, the antihypertrophic ability of fenofibrate may be partially attributed to inhibition of adiponectin and NF-*κ*B.

PPARs are known widely for their lipid metabolism and inflammation-modulating roles. Agonist ligands for PPAR*α* have been clinically used for atherogenic dyslipidemia management [24]. Recently, the pleiotropic effects of PPARs have been noticed with evidence that PPAR*α* is a negative regulator of cardiomyocyte hypertrophy. In addition to its lipid-lowering effect, the PPAR*α* ligand could also be useful for the management of myocardial hypertrophy and remodeling [25–27].

PPAR*α* downregulation is observed in cardiac hypertrophy and heart failure, suggesting that PPAR*α* deficiency can impair heart functional capacity [28–31]. PPAR*α* activator attenuates cardiac dysfunction, cardiac fibrosis, and cardiac hypertrophy; improves survival by regulating redox-regulated transcription factors; and causes suppression of profibrotic, prohypertrophic, and inflammatory genes in animals [28, 32–34]. Previous studies demonstrated that fenofibrate reduces ET-1-caused cardiac hypertrophy by inhibiting AP-1 binding and p38 signaling [7, 8]. Activation of PPAR*α* by fenofibrate can also compete with GATA-4 binding and interfere with ET-1-caused cardiomyocyte hypertrophy [9].

Adiponectin is an insulin sensitizing hormone that exerts its action through its receptors AdipoR1, AdipoR2, and T-cadherin [35]. Since adiponectin is a white and brown adipose tissue hormone, it circulates in the bloodstream in trimeric, hexameric, and high-molecular-mass species, while different forms of adiponectin have been found to play distinct roles in the balance of energy homoeostasis [35]. It also has been reported to play a critical role in cardiac remodeling. By activating AMP-activated protein kinase signal, adiponectin inhibits myocardium hypertrophy [13, 36–39]. Moreover, supplementation of exogenous adiponectin ameliorated cardiac hypertrophy in adiponectin-deficient animals, suggesting that adiponectin may protect cardiomyocytes directly [36–39]. As we know, adiponectin in plasma is increased in patients with overt heart failure; high adiponectin levels can be used as an independent predictor of clinical outcomes in heart failure [40–42]. In our previous study, the adiponectin expression in myocardial tissue was related to the severity of heart failure. Our results provided the first evidence that activated PPAR*α* can decrease activation of adiponectin and NF-*κ*B and inhibit ET-1-induced cardiomyocyte hypertrophy. Thus, adiponectin may play a role in the progression of heart failure and metabolic disturbance in heart failure [42]. However the unremitted increase in plasma levels and myocardial expression of adiponectin in heart failure patients might represent a compensatory response to protect against the myocardial injury progression. However, it might also represent short-term beneficial adaptive responses to acute injury that may eventually turn into harmful maladaptive signals on prolonged and chronic activation, like the sustained sympathetic hyperactivity in the advanced heart failure. Some of the effects of adiponectin may adversely affect the cardiovascular system or even the whole body while being chronically activated. For instance, adiponectin may activate NF-*κ*B and AP-1, resulting in expression of proinflammatory genes and enhancement of angiotensin II-induced cardiac fibroblast proliferation, which may, in turn, play a role in heart failure progression [17, 18]. On the other hand, adiponectin can increase energy expenditure and decrease body weight, which may participate in cardiac cachexia development in advanced heart failure [19, 20].

ET-1 plays a critical role in the progression of myocyte hypertrophy [3, 4]. Various signaling pathways act as downstream effectors of ET-1, including p38 and several NFAT-related signaling systems [7–10]. Recently, ET-1 has been shown as a regulatory factor in secretion of different adipokines, including adiponectin [43, 44]. Through the ETA receptor, ET-1 stimulates adipocytes to secrete adiponectin, suggesting that ET-1 may play a significant role in the regulation of adiponectin in adipose tissue [43, 44]. In our previous studies, we also found that both ET-1 and angiotensin II can acutely stimulate adiponectin expression and significantly increased cell size in cultured HCMs [21, 42]. The use of an angiotensin receptor-blocker can inhibit the angiotensin II-induced adiponectin expression [42, 43]. Therefore, we hypothesized that PPAR*α*, NF-*κ*B, adiponectin, and cardiac hypertrophy may be interrelated. As far as we know, no specific data concerning the interrelation between fenofibrate, ET-1, cell hypertrophy, and adiponectin have yet been reported.

Some limitations are noted in the present study. First, we did not explore if exogenous adiponectin also suppresses ET-1 expression. Second, the current study cannot exclude the possible effects of ET-1 on the regulation of other factors involved in the expression of adiponectin. Moreover, although the mechanism by which NF-*κ*B binding to PPAR*α* decreased their binding to hypertrophic gene promoters remains to be elucidated, it is possible that these molecules could be competing for the same DNA binding site. Furthermore, our present study did not investigate whether fenofibrate may exert its antihypertrophic effect through other mechanisms. Therefore, although our study may provide additional evidence showing the application of PPAR*α* for the treatment of cardiac hypertrophy, further investigation is required to clarify this point [45].

## 5. Conclusions

Our results fit a model in which PPAR*α* activated by fenofibrate can prevent activation of adiponectin and NF-*κ*B, thereby preventing induction of cardiomyocyte hypertrophy by ET-1. Our findings provide a novel mechanistic insight into a role for PPAR*α* and adiponectin in cardiac hypertrophy. We suggest that interference with inflammatory nuclear transcription factors by fenofibrate could be a potential therapeutic approach to prevent cardiac hypertrophy.
